# An Apple a Day: Which Bacteria Do We Eat With Organic and Conventional Apples?

**DOI:** 10.3389/fmicb.2019.01629

**Published:** 2019-07-24

**Authors:** Birgit Wassermann, Henry Müller, Gabriele Berg

**Affiliations:** Institute of Environmental Biotechnology, Graz University of Technology, Graz, Austria

**Keywords:** *Malus domestica*, management practice, plant protection, microbiota, carposphere, edible microbiome, one health concept

## Abstract

Apples are among the most consumed fruits world-wide. They represent a source of direct human exposure to bacterial communities, which is less studied. We analyzed the apple microbiome to detect differences between tissues and the impact of organic and conventional management by a combined approach of 16S rRNA gene amplicon analysis and qPCR, and visualization using fluorescence *in situ* hybridization and confocal laser scanning microscopy (FISH-CLSM). Each apple fruit harbors different tissues (stem, peel, fruit pulp, seeds, and calyx), which were colonized by distinct bacterial communities. Interestingly, fruit pulp and seeds were bacterial hot spots, while the peel was less colonized. In all, approximately 10^8^ 16S rRNA bacterial gene copy numbers were determined in each g apple. Abundances were not influenced by the management practice but we found a strong reduction in bacterial diversity and evenness in conventionally managed apples. In addition, despite the similar structure in general dominated by *Proteobacteria* (80%), *Bacteroidetes* (9%), *Actinobacteria* (5%), and *Firmicutes* (3%), significant shifts of almost 40% of bacterial genera and orders were monitored. Among them, especially bacterial signatures known for health-affecting potential were found to be enhanced in conventionally managed apples. Our results suggest that we consume about 100 million bacterial cells with one apple. Although this amount was the same, the bacterial composition was significantly different in conventionally and organically produced apples.

## Introduction

The host-associated microbiota is involved in health issues of the host; this was shown for humans and plants as well (Derrien and van Hylckama Vlieg, 2015; Berg et al., 2017). Despite being specifically composed and partly deeply embedded within the host, microbial communities are essentially open and interconnected ecosystems (Berg, 2015). However, this connection and the exchange between microbiomes are less understood, despite their importance to health reflected now also in the one health concept (Flandroy et al., 2018). The plant-gut microbiome axis could be of special importance for human health, and raw-eaten plants seem an important source for microbes (Leff and Fierer, 2013; Berg et al., 2014; Wassermann et al., 2017). Recently it was shown that plant-associated microbiota including bacteria, fungi and viruses transiently colonized the gut (David et al., 2014); thus, forming our transient microbiome (Derrien and van Hylckama Vlieg, 2015). However, the microbial diversity associated with vegetables, fruits and herbs is less studied, especially in this context. In contrast, research and rules in this area focus on food-borne pathogens and food safety; food-borne diseases are recognized as a global burden (World Health Organization [WHO], 2015). First microbiome studies suggest that improved understanding of how certain ecologies provide supportive resources for human pathogens on plants, and how components of certain agro-ecologies may play a role in the introduction of human pathogens to plants (Ottesen et al., 2019). However, more knowledge on fresh produce-associated microbiota and a holistic view on the system is crucial for food safety inquiries (Blau et al., 2018).

The plant microbiota play an essential role in plant development and health and exert influence on resilience toward biotic as well as abiotic factors (Berg et al., 2016). In general, the plant microbiota is driven by the plant genotype, differs strongly between below and above ground parts and is affected by soil quality and biotic and abiotic conditions (Berg and Smalla, 2009; Vorholt, 2012; Philippot et al., 2013). While a core plant microbiome is vertically transmitted by seeds, the surrounding environment is another source of the plant microbiota (Berg and Raaijmakers, 2018). Many driving and assembly factors of the plant microbiome are already identified; in agricultural ecosystems management practices have a crucial influence on microbiota composition, diversity and functionality, subsequently affecting health and performance of the host plant (Philippot et al., 2013). Our understanding of the plant microbiome was improved by studies on the model plant *Arabidopsis thaliana* and important crops such as rice and maize (Bulgarelli et al., 2012; Lundberg et al., 2012; Peiffer et al., 2013) but the specific fruit and vegetable microbiome is understudied (Leff and Fierer, 2013). Tomato is a model vegetable for microbiome studies (Bergna et al., 2018; Kwak et al., 2018; Ottesen et al., 2019); in parallel, apples are models for fruit microbiomes.

Apples are among the most consumed fruits world-wide; their production is increasing constantly, and comprise about 83 million t (FAO, 2019). Apples represent the most important dietary source for various flavonoids in our diets, and a beneficial impact on human health due to apple procyanidins and pectin has been frequently described (Shoji and Miura, 2014; Sanz et al., 2015; Shtriker et al., 2018). Studies suggest that apple supplementation can induce substantial changes in microbiota composition and metabolic activity *in vitro*, which could be associated with potential benefits to human health (Koutsos et al., 2017; Garcia-Mazcorro et al., 2019). However, less is known about the apple microbiome; previous work has focused largely on plant pathogens and here, mainly the phyllosphere was studied (Burr et al., 1996; Pusey et al., 2009; Stockwell et al., 2010; Yashiro et al., 2011; He et al., 2012; Liu et al., 2018). Interestingly, apple flowers are colonized by thousands of bacterial taxa, and followed successional groups with coherent dynamics whose abundances peaked at different times before and after bud opening (Shade et al., 2013). The fungal community associated with the apple endosphere is pedigree-specific (Liu et al., 2018), and significantly dependent on different tissues (stem end, calyx end, peel, and wounded flesh) within the apple carposphere (Abdelfattah et al., 2016). However, basic insights into the bacterial communities of apple fruits are still missing.

The objective of this study are basic insights into the apple fruit microbiome. In detail, we aim to identify (i) differences between tissues of apple fruits and (ii) the impact of organic and conventional management practices – which represent diverse defined abiotic treatments pre- and post-harvest – on abundance and composition of apple fruit-associated bacteria. We hypothesize (i) that each apple provides different niches for bacterial communities and (ii) that the management practice has substantial impact on the apple microbiome, which is crucial for plant (post-harvest) and human health issues. With our experimental design we targeted to decipher to which microbiota the consumer is usually directly exposed, and used an integrated design of methods combining 16S rRNA amplicon libraries and qPCR and FISH-CLSM.

## Materials and Methods

### Sampling and Experimental Design

In order to investigate and compare the microbiome of organically and conventionally managed apples (*Malus pumila* Mill.) the cultivar “Arlet” was selected. Both the organically and the conventionally produced apples were cultivated in Styria (Austria) under AMAG.A.P. Certification (AMA-Gütesiegel-Produktion), which represent the Austrian law for the international guidelines for agricultural management program GLOBALG.A.P. Matured, fully developed apples were sampled at harvest time in September 2017 in Styria (Austria). Organically managed apples originated from an organic orchard, which follows the international “demeter” guidelines for organic farming^1^, using sterile gloves and instruments. Conventional apples originated from a conventional orchard in Styria. In contrast to the organically produced apples, they underwent the following post-harvest treatments: directly after harvest, apples were short-term stored under controlled atmosphere (1–2°C, 1.5–2% CO_2_), washed and wrapped in polythene sheets for sale. Both apple management groups (“organic” and “conventional”) were transported to laboratory immediately and processed under sterile conditions. All apples were visually examined for consistency in shape, size, color, flawlessness, firmness, and freshness prior to processing. Four apples, weighing 190 ± 5 g, were selected from each of the two management groups and each apple was divided into six tissues with the following weights: stem: 0.2 g, stem end: 2 g, peel: 9 g, fruit pulp: 12 g, seeds: 0.2 g, and calyx end: 3 g. Thus, each tissue was represented by four replicates, where each replicate consists of the respective tissue of one apple. Here it has to be mentioned that seeds of conventionally managed apples contained on average only half as many seeds as organically managed ones.

### Microbial DNA Extraction and Amplicon Library Construction

In order to extract microorganism, stem end, peel, fruit pulp and calyx end samples were homogenized in a Stomacher laboratory blender (BagMixer, Interscience, Saint-Nom-la-Bretèche, France) with 4 ml sterile NaCl (0.85%) solution for 3 min. Seeds and stems were physically disrupted in a sterilized mortar. For the upcoming cultivation-independent analyses, 2 ml of apple suspensions were centrifuged for 20 min at 16,000 *g* and pellets were used to extract bacterial genomic DNA using FastDNA SPIN Kit for Soil (MP Biomedicals, Solon, OH, United States) and a FastPrep Instrument (MP Biomedicals, Illkirch, France) for 30 s at 5.0 m/s. For culture-independent Illumina MiSeq v2 (250 bp paired end) amplicon sequencing, the primers 515f – 806r (Caporaso et al., 2010) were used to amplify the 16S rRNA gene using three technical replicates per sample. Peptide nucleic acid (PNA) clamps were added to PCR mix to block amplification of host plastid and mitochondrial 16S DNA (Lundberg et al., 2013). PCR for 16S rRNA gene amplification was performed in a total volume of 30 μl [5× Taq&Go (MP Biomedicals, Illkirch, France), 1.5 μM PNA mix, 0.25 mM of each primer, PCR-grade water and 1 μl template DNA] under the following cycling conditions: 95°C for 5 min, 30 cycles of 96°C for 1 min, 78°C for 5 s, 54°C for 1 min, 74°C for 60 s and a final elongation at 74°C for 10 min. Technical replicates were pooled and purified by Wizard SV Gel and PCR Clean-Up System (Promega, Madison, WI, United States). For amplicon sequencing, DNA concentrations were measured with Nanodrop 2000 (Thermo Fisher Scientific, Wilmington, DE, United States) and samples were combined in equimolar concentration.

### Illumina MiSeq Data Analysis and Statistics

Raw sequence data preparation and data analysis was performed using QIIME 1.9.1 (Caporaso et al., 2010). After paired reads were joined and quality filtered (phred q20), chimeric sequences were identified using usearch7 (Edgar, 2010) and removed. Representative sequences were aligned, open reference database SILVA (ver128_97_01.12.17) was used to pick operational taxonomic units (OTUs) and *de novo* clustering of OTUs was performed using usearch. After taxonomy assignment, sequences assigned to host mitochondria and chloroplasts were discarded. OTU tables were rarefied to 1,525 sequences per sample, according to the sample with lowest amount of sequences. Rarefied OTU tables served as input matrix for upcoming alpha and beta diversity analyses and according statistics were calculated in QIIME. Beta diversity, based on unweighted UniFraq distance matrix, was visualized by Principle Coordinates Analysis (PCoA) and statistical significance was calculated by Analysis of Similarity (ANOSIM). Box-and-Whiskers-Plots, based on Shannon diversity indices, were constructed to visualize microbiota diversity of apple samples using IBM SPSS program (version 25.0, IBM Corporation, Armonk, NY, United States) and statistics were calculated using non-parametric Kruskal–Wallis test and False Discovery Rate (FDR) multiple test correction. For taxonomy charts and in order to trace differentially abundant taxa between organically and conventionally managed apples, OTUs with less than 0.01% abundance were excluded from the dataset. Significant differences (α < 0.05) in taxa abundance on genus and order level were calculated in QIIME, using non-parametric Kruskal–Wallis/FDR test. Taxonomy charts were constructed by merging the core microbiota (taxa occurring in 75% of all replicates) of each tissue of the corresponding management group and the taxonomic network was constructed using Cytoscape version 3.5.1 (Shannon et al., 2003).

### Quantitative PCR (qPCR)

For determining bacterial abundance, qPCRs were conducted with the bacterial directed primer pair 515f – 927r (10 μm each; Köberl et al., 2011). The qPCR reaction mix contained 5 μl KAPA SYBR Green, 0.15 μl PNA mix, 0.5 μl of each primer, 2.85 μl PCR-grade water, and 1 μl template DNA (fruit pulp and seed samples were diluted 1:10 in PCR grade water). Quantification of fluorescence was detected in a Rotor-Gene 6000 real-time rotary analyzer (Corbett Research, Sydney, Australia) with the following cycling conditions: 95°C for 5 min, 40 cycles of 95°C for 20 s, 54°C for 30 s, 72°C for 30 s and a final melt curve of 72 to 96°C. Three individual qPCR runs with *R*^2^-values of standard curves of 0.12 were conducted separately and each replicate was measured in triplicate. Intermittently occurring gene copy numbers that were detected in negative control reactions were subtracted from the respective sample. Significant differences (*p* < 0.05) of bacterial gene copy numbers per gram of tissue between management groups and apple tissues were calculated using IBM SPSS program by applying non-parametric Kruskal–Wallis test including FDR multiple test correction.

### Fluorescent *in situ* Hybridization (FISH) and Confocal Laser Scanning Microscopy (CLSM)

Native colonization patterns of bacteria associated with the apple tissues were visualized by FISH-CLSM, using a Leica TCS SPE confocal laser scanning microscope (Leica Microsystems, Mannheim, Germany) with oil immersion objective lenses Leica ACS APO 40.0× oil CS and Leica ACS APO 63× oil CS. Apple samples were fixed with 4% paraformaldehyde/phosphate-buffered saline over-night at 4°C prior to FISH application, according to the protocol of Cardinale et al. (2008). Cy3-labeled EUB338MIX (Daims et al., 1999; Amann et al., 2001) was used to stain overall bacterial colonization and for specific visualization of *Firmicutes* and *Gammaproteobacteria*, Cy5-labeled LGC-mix (Meier et al., 1999) and ALEXA-labeled GAM42a (Manz et al., 1992), respectively, were applied. For contrasting host cell walls, FISH samples were treated with Calcoflour White. By maximum projections of optical z-stack slices, micrographs of the bacterial colonization were generated.

## Results

### Quantitative Records of Bacterial 16S rRNA Gene Abundance in Apple Tissues

**FIGURE 1:**
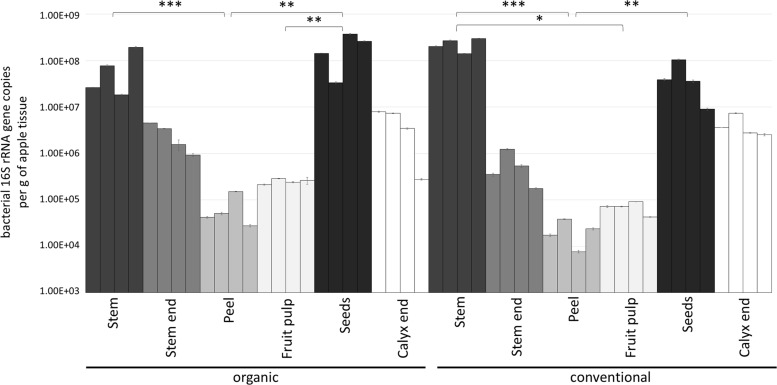
Bacterial abundance in carposphere tissues of organically and conventionally managed apples. Microbial community abundance within each tissue was measured in four replicates by qPCR using PNAs to block mitochondrial and plastid 16S DNA. Asterisks indicate significant differences in 16S rRNA gene abundance (calculated per g of apple tissue) between the tissues within a management group.

Gene copy numbers of bacterial 16S rRNA per gram tissue of organic and conventional apples were measured by qPCR inquiry (Figure 1). Bacterial abundances were observed to be mostly consistent between the management analogs of each tissue; no significant differences (*p* < 0.05) were observed according to non-parametric Kruskal–Wallis/FDR. In contrast, bacterial abundance was strongly tissue-specific. Overall, stem (mean value 1.54 × 10^8^ 16S rRNA gene copy numbers per gram) and seeds (mean value 1.26 × 10^8^) showed highest bacterial abundance, followed by calyx end, stem end and fruit pulp; peel microbiota (mean value 4.49 × 10^4^) were lowest abundant. Table 1, therefore, shows only the significant difference in 16S rRNA gene abundance per gram between the tissues within the two management groups. Combining all tissue samples of the corresponding management group resulted in the mean values 4.85 × 10^7^ and 4.67 × 10^7^ per gram organic and conventional apple, respectively. The difference was not significant. In order to give a notion on the amount of bacteria ingested during the consumption of a whole apple, we excluded stem samples and multiplied the values of 16S rRNA gene copy numbers per g tissue with the mean weight of the respective tissue within one “Arlet” apple: stem end: 6 g, peel: 35 g, fruit pulp: 145 g, seeds: 0.3 g, calyx end: 5 g. Calculated values were then added up; accordingly, consumption of one organic and one conventional “Arlet” apple includes ingestion of 1.39 × 10^8^ and 4.19 × 10^7^ 16S rRNA gene copy numbers, respectively. If you eat only peel and fruit pulp, 3.87 × 10^7^ and 3.39 × 10^6^ 16S rRNA gene copies are ingested with one organic and one conventional apple, respectively. The differences were not statistically significant. “Arlet” apples represent a relatively small apple variety; considering the standard size of an apple with 240 g, consuming the whole apple includes a mean uptake of 1.14 × 10^8^ 16S rRNA gene copy numbers.

**TABLE 1:** Significant differences in 16S rRNA gene abundance per gram of tissue between organically and conventionally managed apple tissues.

	Group1^*^	Group2^*^	**Group1 mean**	**Group2 mean**	***p*-Value**
Organic tissues	Stem O	Peel O	7.91E+07 ± 6.99E+07	6.81E+04 ± 4.89E+04	0.001
	Peel O	Seeds O	6.81E+04 ± 4.89E+04	2.04E+08 ± 1.28E+08	0.002
	Fruit pulp O	Seeds O	2.51E+05 ± 2.80E+04	6.81E+04 ± 1.28E+08	0.004
Conventional tissues	Seeds C	Peel C	4.71E+07 ± 3.50E+07	2.18E+04 ± 1.12E+04	0.002
	Stem C	Peel C	2.28E+08 ± 6.16E+07	2.18E+04 ± 1.12E+04	0.001
	Stem C	Fruit pulp C	2.28E+08 ± 6.16E+07	6.96E+04 ± 1.76E+04	0.02

^*^O and C denote for organically and conventionally managed apples, respectively. Only significant differences in microbial abundance between apple tissues are listed.

### Quantitative Records of Diversity Estimates of Apple Microbiota

**FIGURE 2:**
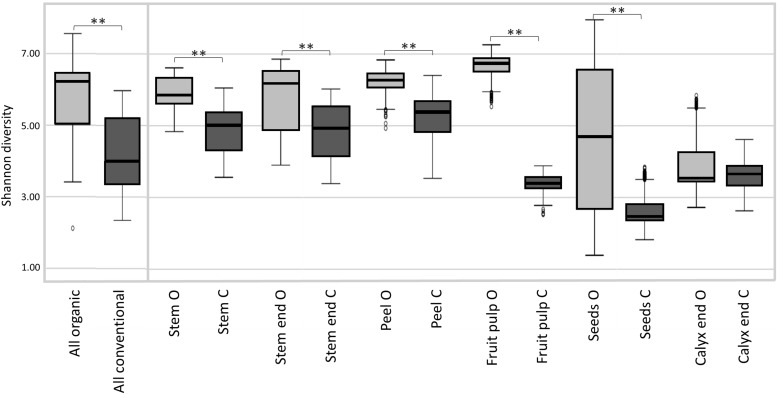
Microbial diversity estimates of organically and conventionally managed apples and apple tissues. Suffixes O and C of carposphere tissue in the bottom legend, denote for organic and conventional management, respectively. Significant differences in Shannon diversity estimates of the apple management analogs are indicated by brackets and asterisks.

Shannon diversity estimates revealed organically managed apples to harbor a significantly more diverse microbiota than conventionally managed ones (Figure 2 and Table 2). The difference was even more significant when the two management analogs of each tissue were compared; Shannon diversity index was significantly higher for the microbiota of all organic tissues, compared to conventional ones, with the sole exception of calyx end microbiota. Table 2 shows furthermore the comparison of the tissues within one management group. For organic apples, fruit pulp showed highest microbial diversity, followed by peel and stem, stem end, seed and calyx end, in ascending order. Diversity of the fruit pulp microbiota was significantly higher than stem, seeds and calyx end microbiota. Regarding conventional tissues, Shannon diversity index was highest for peel microbiota, followed by stem, stem end, fruit pulp, calyx end, and seed microbiota. Here, peel microbiota was significantly more diverse than seed, calyx end, and fruit pulp microbiota.

**TABLE 2:** Alpha diversity measures of differentially managed apples and apple tissues based on Shannon diversity estimates.

	Group1^*^	Group2^*^	**Group1 mean**	**Group2 mean**	***p*-Value^∗∗^**
Whole apple	All organic	All conventional	5.60±1.36	4.17±1.11	0.003
Organic vs. conventional tissues	Stem end O	Stem end C	5.87±0.81	4.94±0.71	0.001
	Stem O	Stem C	5.92±0.41	4.98±0.64	0.001
	Peel O	Peel C	6.22±0.32	5.32±0.57	0.001
	Fruit pulp O	Fruit pulp C	6.67±0.35	3.39±0.25	0.001
	Seeds O	Seeds C	4.97±2.13	2.68±0.50	0.001
	Calyx end O	Calyx end C	3.96±0.87	3.70±0.47	0.782
Organic tissues	Peel O	Stem end O	6.22±0.32	5.87±0.81	1
	Peel O	Stem O	6.22±0.32	5.92±0.41	1
	Peel O	Seeds O	6.22±0.32	4.97±2.13	0.157
	Peel O	Calyx end O	6.22±0.32	3.96±0.87	0.002
	Peel O	Fruit pulp O	6.22±0.32	6.67±0.35	0.157
	Stem end O	Stem O	5.87±0.81	5.92±0.41	0.157
	Stem end O	Seeds O	5.87±0.81	4.97±2.13	0.002
	Stem end O	Calyx end O	5.87±0.81	3.96±0.87	0.001
	Stem end O	Fruit pulp O	5.87±0.81	6.67±0.35	1
	Stem O	Seeds O	5.92±0.41	4.97±2.13	1
	Stem O	Calyx end O	5.92±0.41	3.96±0.87	0.175
	Stem O	Fruit pulp O	5.92±0.41	6.67±0.35	0.002
	Seeds O	Calyx end O	4.97±2.13	3.96±0.87	1
	Seeds O	Fruit pulp O	4.97±2.13	6.67±0.35	0.001
	Calyx end O	Fruit pulp O	3.96±0.87	6.67±0.35	0.001
Conventional tissues	Peel C	Stem end C	5.32±0.57	4.94±0.71	1
	Peel C	Stem C	5.32±0.57	4.98±0.64	0.157
	Peel C	Seeds C	5.32±0.57	2.68±0.50	0.001
	Peel C	Calyx end C	5.32±0.57	3.70±0.47	0.001
	Peel C	Fruit pulp C	5.32±0.57	3.39±0.25	0.001
	Stem end C	Stem C	4.94±0.71	4.98±0.64	1
	Stem end C	Seeds C	4.94±0.71	2.68±0.50	0.001
	Stem end C	Calyx end C	4.94±0.71	3.70±0.47	0.003
	Stem end C	Fruit pulp C	4.94±0.71	3.39±0.25	0.116
	Stem C	Seeds C	4.98±0.64	2.68±0.50	0.002
	Stem C	Calyx end C	4.98±0.64	3.70±0.47	0.209
	Stem C	Fruit pulp C	4.98±0.64	3.39±0.25	1
	Seeds C	Calyx end C	2.68±0.50	3.70±0.47	1
	Seeds C	Fruit pulp C	2.68±0.50	3.39±0.25	0.209
	Calyx end C	Fruit pulp C	3.70±0.47	3.39±0.25	1

^*^O and C denote for organically and conventionally managed apples, respectively. ^∗∗^Statistics were calculated based on Kruskal–Wallis/FDR test.

**FIGURE 3:**
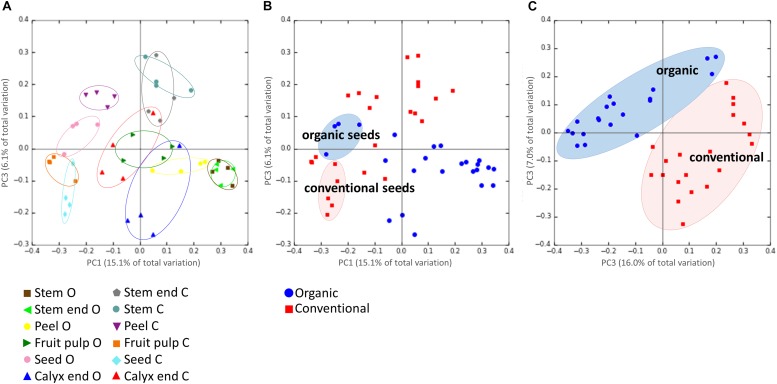
Beta-diversity analysis on microbiota composition dependencies. Panel **(A)** shows the microbiota composition grouped by the tissue of the respective management group, where O and C in the bottom legend denote for organically and conventionally managed apples, respectively. Panel **(B)** visualizes composition of all tissue replicates, colored by organic (blue circles) and conventional (red squares); seeds of organically and conventionally managed apples are highlighted. In Panel **(C)**, same dataset is shown but seed samples of both management groups were excluded. PCoA plots are based on unweighted UniFraq distance matrix.

Highest beta diversity measures were observed when the replicates were grouped by the tissue of the respective management group (ANOSIM values: *R* = 0.8, *p* = 0.001; Figure 3A). Grouping samples by organic and conventional management revealed the ANOSIM values *R* = 0.26, *p* = 0.001 (Figure 3B). Hence, we had a closer look on the management effect on each tissue separately, resulting in the ANOSIM values *R* > 0.8, *p* < 0.05 for all tissues, except seeds (ANOSIM values for seeds: *R* = 0.4, *p* = 0.05). The management practice therefore seems to have a profound impact on the microbiota composition of all tissues while the management effect on seed microbiota was lower. This observation was confirmed when seed samples were excluded from the dataset; ANOSIM values increased to *R* = 0.45 and *p* = 0.001 (Figure 3C).

### The General Structure of the Bacterial Apple Microbiota

After removing chimeric, mitochondrial and chloroplast sequences, the overall bacterial community of all apple samples, assessed by 16S rRNA gene amplicon sequencing, contained 6,711,159 sequences that were assigned to 92,365 operational taxonomic units (OTUs). The taxonomic assignment of OTUs revealed 44 different phyla, 325 orders and 1,755 genera. Among bacterial phyla, *Proteobacteria* highly dominated with 80%, followed by *Bacteroidetes* (9%), *Actinobacteria* (5%), and *Firmicutes* (3%). *Burkholderiales* were highly abundant concerning bacterial orders (31% abundance), followed by *Sphingomonadales* (14%), *Rhizobiales* (12%), *Pseudomonadales* (11%), *Enterobacteriales* (7%) and *Cytophagales* (5%); *Micrococcales*, *Sphingobacteriales*, *Bacillales, Rhodospirillales*, and *Flavobacteriales*, in ascending order, represented between 5 and 1% of total OTUs. OTUs assigned to the genus *Ralstonia* were most frequent with 13%, while *Sphingomonas* (12%), *Pseudomonas* (11%), *Massilia* (7%), *Methylobacterium* (7%), *Burkholderia* (5%), *Pantoea* (5%), and *Hymenobacter* (5%) were furthermore high abundant.

### The Specific Structure of the Microbiota in Tissues of Organic and Conventional Apples

**FIGURE 4:**
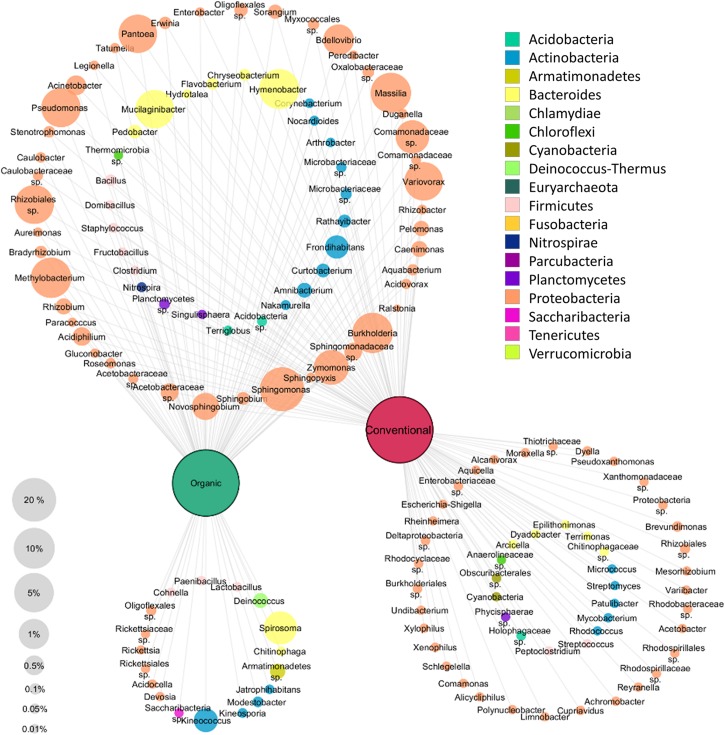
Core and specific microbiota for organic and conventional apples. Core microbiota (taxa occurring in 75% of all replicates) of each management group (conventional and organic) were combined for network analysis. To be included, taxa had to exhibit at least 0.01% abundance in the whole dataset. Node size correspond to relative abundance in the dataset as denoted in the legend on the bottom left, node labels display taxonomic identification of OTUs on genus level wherever possible and node color indicates appropriate phylum, as described in the legend on the top right.

A clustering network based on the core taxa of the tissues of each apple management group was constructed to visualize the taxa present in all apples as well as the taxa that are specific for each management group (Figure 4). Only taxa occurring with at least 0.01% abundance in the whole dataset were included in the network analysis. All apples were found to share a high abundant core microbiota; 73 out of 141 genera were shared. Among them, highly abundant *Proteobacteria* were most dominant and abundant with 45 genera. In total 16 genera were found only in organically managed apples, and 50 genera, predominated by *Proteobacteria* (33 genera) were specific for conventional apples. Overall, the specific microbiota for each management group were less abundant than the shared microbiota.

**FIGURE 5:**
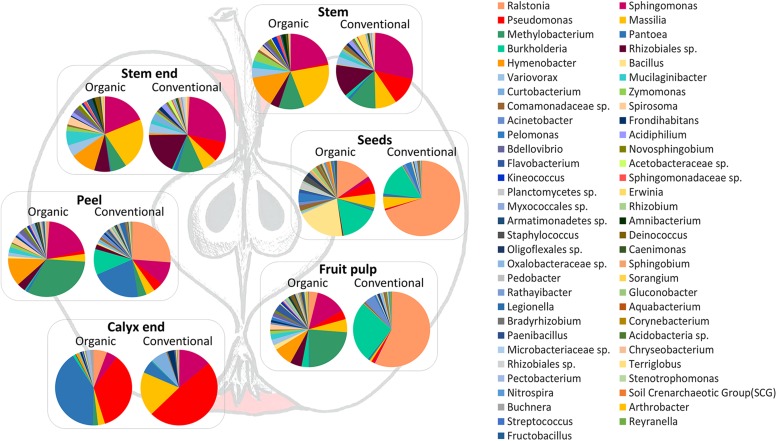
Taxonomic composition of organic and conventional apple tissue microbiota. Pie charts visualize taxa occurring in the core microbiomes of each tissue, with at least 0.1% abundance in the whole dataset, and visualize differences between conventional and organic apples.

In order to visualize the differences between the community compositions of the management analog of each tissue on a taxonomic level, Figure 5 was prepared. Pie charts include only taxa that are abundant with at least 0.1% in the whole dataset. Here, differences between organically and conventionally managed apples are obvious for all tissues. Contradictory to beta diversity analysis (described above and Figure 3), seeds appear to feature very different microbiota, especially due to the dominance of *Ralstonia* in conventional seeds. The inconsistency of the results can be explained by the fact that beta diversity measures were calculated on the entire OTU table and Figure 5 was constructed on the high abundant (>0.1%) core taxa of each tissue.

### Indicator Species for Organically and Conventionally Managed Apples

Differences in abundance of specific bacterial taxa associated with either organically or conventionally managed apples, were assessed by applying non-parametric Kruskal–Wallis/FDR test. Priorly, OTU table was filtered by excluding OTUs with less than 0.01% abundance, resulting in a total of 172 taxa on genus level. Calculations assigned 67 taxa a significantly higher abundance in either organically or conventionally managed apples (Supplementary Table 1); accordingly, 39% of the taxa were significantly different abundant. Noteworthy among them are *Methylobacterium*, *Hymenobacter*, *Spirosoma*, and *Zymomonas* which were high abundant in organically managed apples, and *Burkholderia*, *Pantoea*, *Erwinia*, and *Acinetobacter*, especially high abundant in conventional apples. Significantly different abundance between microbiota of organically and conventionally managed apples was furthermore calculated on higher taxonomic level. The 172 genera were condensed to 66 different bacterial orders; among them, 25 orders were significantly different abundant, accounting to 37.8% (Supplementary Table 1). Among those, *Cytophagales* were high abundant in organic apples while the orders *Burkholderiales*, *Pseudomonadales*, *Enterobacteriales*, and *Flavobacteriales* prevailed in conventional apples.

### Indicator Species for Health With Focus on *Enterobacteriales*

**FIGURE 6:**
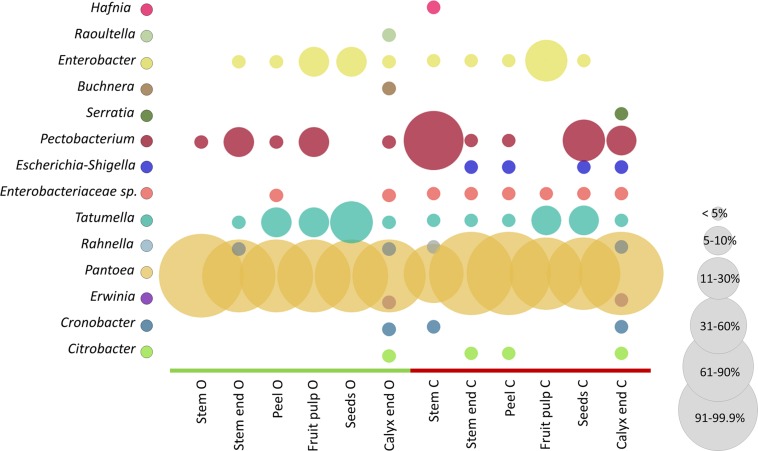
Comparison of conventional and organic apple tissues regarding *Enterobacteriales* abundance. Color code for bubbles is depicted in the legend on the left and bubble size indicates relative abundance of taxa within total *Enterobacteriales* microbiota, as explained in the legend on the right. The abbreviations O and C denote for organically and conventionally managed apple tissues, respectively.

The microbiota of conventional and organic apples were screened for their potential to feature health-relevant properties for humans. For that purpose, we constructed an OTU table containing only *Enterobacteriales*, as especially this order is described to contain taxa responsible for food-borne outbreaks. In our dataset the order *Enterobacteriales* was found to be significantly more abundant in conventionally managed apples (described above and Supplementary Table 1). Figure 6 shows the relative abundance of taxa to total *Enterobacteriales* in the tissues of organically and conventionally managed apples. *Pantoea* was most abundant among all samples, representing between 60 and 99% of *Enterobacteriales* microbiota; however, *Pantoea* was significantly more abundant in conventionally managed apples (Supplementary Table 1). *Pectobacterium*, *Tatumella*, and *Enterobacter* were furthermore abundant in almost all tissues, independent of their management practice. Abundance of a not further assigned *Enterobacteriaceae* taxon (*Enterobacteriaceae* sp. in Figure 6), *Erwinia* and *Escherichia-Shigella* were significantly more abundant in conventional apples.

### Native Colonization Patterns of Microbiota in Apple Tissues

**FIGURE 7:**
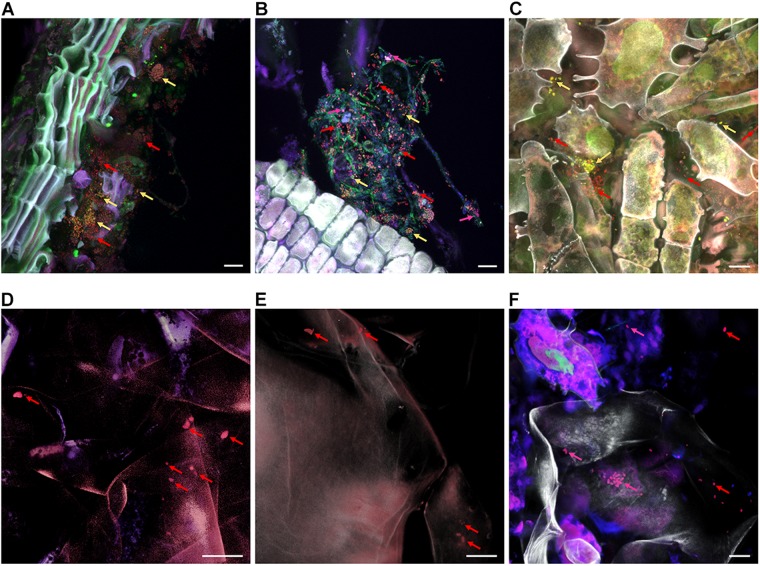
FISH-CLSM micrographs showing bacterial colonization of organic apple tissues. Panels **(A–F)** visualize stem, stem end, peel, fruit pulp, seeds and calyx end samples, respectively. Bacteria were stained with FISH probes specific for *Gammaproteobacteria* (fluorescing pink and indicated by pink arrows), *Firmicutes* (yellow) and remaining bacteria of other classes (red); host structures are fluorescing white. Bar on the bottom right of each panel denotes for 10 μm.

By using CLSM in combination with FISH we were able to visualize bacteria native to all carposphere tissues *in situ* (Figure 7). Visualization of stem, stem end, peel, and calyx end microbiota turned out to be successful; *Gammaproteobacteria* (fluorescing pink) and *Firmicutes* (yellow) were distinguishable from remaining bacteria (red). In fruit pulp and seed samples, few bacteria were detected as well, however, due to high autofluorescence of host tissues, imaging was more challenging compared to remaining tissues. During microscopic observations, no differences were observed between organic and conventional apples, therefore Figure 7 illustrates only tissues of organic apples.

## Discussion

In the present study we identified tissue-specific and management-specific microbial communities for apple fruits. This specificity was apparent for all tissues regarding both microbiota composition and diversity, but not abundance. The observed differences between organic and conventional apples could certainly be attributable to a variety of factors within farming and storage conditions.

Deciphering the bacterial microbiota of Austrian “Arlet” apples resulted in a drastic diversification between the six tissues within the apple carposphere for bacterial abundance, diversity and composition. Interestingly, alpha diversity estimates and calculations of bacterial abundance (according to qPCR) were pretty much inconsistent. Whereas fruit pulp and peel featured highest values for diversity, microbiota abundance was lowest in those tissues. Seeds, on the other hand, were less divers than other tissues, but showed highest abundance. Those results were partially confirmed by FISH-CLSM; high microbial abundance was visualized on stem, stem end and calyx end samples, whereas peel and fruit pulp turned out to be less colonized. However, for seeds it was not possible to visualize the high bacterial abundances indicated by qPCR which was due to exceptionally high autofluorescence in seed tissues. Differences between the tissue-associated microbiota were expected beforehand, as varying metabolic and nutrient conditions in the specific parts are certain. The sole responsibility of all the different parts of a fruit is to protect the seeds and enable their dispersal for a successful reproduction of the plant. Apple seed microbiota showed interesting features: among all tissues, seeds, together with stem, were found to significantly prevail in bacterial quantity, hosting an average of 126 billion bacterial gene copy numbers per gram seeds. Seed microbiota composition was most similar to fruit pulp microbiota which underline the vertical microbiome transmission in plants (Hardoim et al., 2012).

The management practice was found to significantly drive the microbiota of all tissues within the apple. Diversity was significantly higher in all organically grown tissues (except for calyx end) and the microbiota composition was distinct between organic and conventional tissue analogs. Compared to the other tissues, seed microbiota was lowest affected by the management practice, while the exclusion of low abundant taxa from the dataset resulted in dramatic dissimilarities between organic and conventional seeds. Organic seeds showed a much more even composition than conventional seeds which were highly dominated by *Ralstonia*. Altogether, organic apple microbiota was significantly more divers and differentially composed; the remarkable amount of 39% of genera and 38% of bacterial orders was significantly different abundant. Referring to a previous work on the apple flower microbiome, *Deinococcus-Thermus* and *Saccharibacteria* (formally known as TM7) dominated the community (Shade et al., 2013). In the present study, both taxa were present in almost all replicates of organic apples (0.6 and 0.08%, respectively), in contrast to conventional ones (0.01% *Deinococcus-Thermus* and 0.007% *Saccharibacteria*). This promotes exceptional specificity and functionality of the microbiota for successive development stages from the flower to the mellow fruit and potentially suggests organic management to rather allow the formation of a stable and beneficial community. Conventional apple microbiota was furthermore found to be less even constructed and highly dominated by *Burkholderiales*, accounting to almost 43% abundance. The order *Enterobacteriales* was one of the signature taxa of conventional apples as well; among them, we would like to highlight the almost ubiquitous occurrence of OTUs assigned to *Escherichia-Shigella* in the tissues of conventional apples (although low abundant) and their absence in organically managed apples. Higher abundances of *Enterobacteriales* in conventional fresh produces compared to organic equivalents have already been reported by Leff and Fierer (2013). Controversially, *Lactobacillus*, which is frequently used within probiotics (Derrien and van Hylckama Vlieg, 2015), was one of the core taxa of organic apples. The highly diverse microbiome of organically managed apples might probably limit or hamper the abundance of human pathogens, simply by outcompeting them; negative correlations between human pathogen abundance and the natural microbiome of fresh produce has already been described (Cooley et al., 2006). The described microbial patterns in organic apples resemble the impact of apple polyphenols on human health, which have not only been shown to alleviate allergic symptoms (Zuercher et al., 2010), but also to promote growth of *Lactobacillus* and *Bifidobacterium* in the human gut and to reduce abundance of food-borne pathogens (Taguri et al., 2004; Bialonska et al., 2010). Considering that specific microbial signatures have potential to reduce food allergies (Kalliomäki et al., 2010), the native microbiome of organic and unprocessed apples could be an advantageous tool to manage and prevent allergic diseases. *Methylobacterium*, identified to enhance the biosynthesis of strawberry flavor compounds (Verginer et al., 2010), was significantly higher abundant in organic apples; here especially on peel and fruit pulp samples. In contrast, *Ralstonia* and *Erwinia*, frequently described for adverse impact on plant health (Denny, 2007; Pirhonen et al., 2018), prevailed in conventional apples. Our results are in significant accordance to a recent study on the apple fruit-associated fungal community (Abdelfattah et al., 2016), where the authors observed specificity of the fungal microbiota to different tissues and management practices. Concordantly, the management practice is suggested to be accountable for the different bacterial and fungal community composition. The lowest effect was observed on seed microbiota, which is mainly cultivar-driven (Berg and Raaijmakers, 2018).

Calculations of 16S rRNA gene abundance resulted in significant differences between tissues but not for the management. This suggests bacteria to occupy the tissues of organically and conventionally produced apples in a similar quantity, while the management practice drives composition and diversity. For the quantitative analyses we used PNAs to block amplification of 16S rRNA of host origin; nevertheless, there is still a possibility that non-bacterial 16S rRNA genes are amplified. Furthermore, qPCR results do not exclusively represent the viable bacterial community. However, comparing gene abundances between tissues and management groups is possible and reliable in this regard.

## Conclusion

Investigating the apple fruit microbiota resulted in profound differences between the tissues, applicable for microbiota diversity, composition and abundance. A significant management effect on the microbiota was furthermore apparent for all tissues, even for seeds. Organic and conventional apples are occupied by a similar quantity of microbiota; consuming the whole apple includes an approximate uptake of 100 million bacterial gene copy numbers. However, freshly harvested, organically managed apples harbor a significantly more diverse, more even and distinct microbiota, compared to conventional ones; the abundance of almost 40% of bacterial genera and orders differed significantly between organically and conventionally managed apples. Moreover, organic apples conceivably feature favorable health effects for the consumer, the host plant and the environment in contrast to conventional apples, which were found to harbor potential food-borne pathogens.

## Data Availability

The raw sequence files supporting the findings of this manuscript are available from the European Nucleotide Archive (ENA) at the study Accession Number: PRJEB32455.

## Author Contributions

BW performed the experiments, analyzed the data, and wrote the manuscript. HM analyzed the data. GB designed the study, discussed the results, and wrote the manuscript. All authors read and approved the final version of the manuscript.

## Conflict of Interest Statement

The authors declare that the research was conducted in the absence of any commercial or financial relationships that could be construed as a potential conflict of interest.

## Footnotes

1 https://www.demeter.at/richtlinien/
