# Supplementary material for: An Apple a Day: Which Bacteria Do We Eat With Organic and Conventional Apples?
Source: Front Microbiol. 2019 Jul 24;10:1629. doi: 10.3389/fmicb.2019.01629 (PMC6667679; doi:10.3389/fmicb.2019.01629)
Supplement: Supplementary file 1 [file Table_1.DOCX]

***Supplementary Material***

**Supplementary Table 1:** Bacterial taxa on genus and order level with significantly different abundance between organically and conventionally managed apples.

| **Organic** | **Conventional** | **Bacterial genera** | **Organic** | **Conventional** | **Bacterial orders** |
| --- | --- | --- | --- | --- | --- |
| **6.76** | 3.25 | Methylobacterium | **7.11** | 0.45 | Cytophagales |
| **5.97** | 0.21 | Hymenobacter | **0.83** | 0.08 | Bdellovibrionales |
| **1.04** | 0.01 | Spirosoma | **0.62** | 0.02 | Kineosporiales |
| **0.99** | 0.24 | Zymomonas | **0.51** | 0.00 | Deinococcales |
| **0.77** | 0.06 | Bdellovibrio | **0.48** | 0.01 | Armatimonadetes p. |
| **0.57** | 0.02 | Kineococcus | **0.31** | 0.03 | Frankiales |
| **0.48** | 0.00 | Deinococcus | **0.25** | 0.02 | Nitrosomonadales |
| **0.48** | 0.01 | Armatimonadetes sp. | **0.12** | 0.10 | Clostridiales |
| **0.44** | 0.08 | Amnibacterium | 16.71 | **42.67** | Burkholderiales |
| **0.26** | 0.04 | Paenibacillus | 5.61 | **13.27** | Pseudomonadales |
| **0.25** | 0.04 | Sorangium | 5.19 | **5.41** | Enterobacteriales |
| **0.16** | 0.00 | Rickettsiaceae sp. | 0.56 | **1.09** | Flavobacteriales |
| **0.11** | 0.00 | Oligoflexales sp. | 0.19 | **0.19** | Rhodobacterales |
| **0.10** | 0.01 | Modestobacter | 0.00 | **0.12** | Holophagales |
| **0.10** | 0.01 | Cohnella | 0.06 | **0.11** | Obscuribacterales |
| **0.10** | 0.00 | Chitinophaga | 0.04 | **0.08** | Rhodocyclales |
| **0.06** | 0.00 | Rickettsiales sp. | 0.01 | **0.05** | Deltaproteobacteria p. |
| **0.06** | 0.00 | Nakamurella | 0.02 | **0.04** | Oceanospirillales |
| **0.05** | 0.00 | Kineosporia | 0.00 | **0.04** | Cyanobacteria p. |
| **0.04** | 0.04 | Variibacter | 0 | **0.04** | Chromatiales |
| **0.02** | 0.00 | Anaerococcus | 0.00 | **0.03** | Chlamydiales |
| **0.02** | 0.00 | Rhodanobacter | 0.00 | **0.03** | Pseudonocardiales |
| 1.55 | **8.26** | Burkholderia | 0.00 | **0.03** | Thiotrichales |
| 3.79 | **3.96** | Pantoea | 0.00 | **0.02** | Anaerolineales |
| 0.47 | **1.26** | Erwinia |  |  |  |
| 0.26 | **1.24** | Acinetobacter |  |  |  |
| 0.01 | **0.47** | Rhizobiales sp. |  |  |  |
| 0.00 | **0.18** | Brevundimonas |  |  |  |
| 0.10 | **0.16** | Chryseobacterium |  |  |  |
| 0.06 | **0.16** | Reyranella |  |  |  |
| 0.00 | **0.12** | Holophagaceae sp. |  |  |  |
| 0.06 | **0.11** | Obscuribacterales sp. |  |  |  |
| 0.04 | **0.11** | Caulobacter |  |  |  |
| 0.04 | **0.10** | Moraxella |  |  |  |
| 0.00 | **0.10** | Arcicella |  |  |  |
| 0.01 | **0.07** | Rhizobacter |  |  |  |
| 0.00 | **0.07** | Dyella |  |  |  |
| 0.04 | **0.06** | Micrococcus |  |  |  |
| 0.01 | **0.06** | Rhodospirillales sp. |  |  |  |
| 0.00 | **0.05** | Deltaproteobacteria sp. |  |  |  |
| 0.00 | **0.04** | Rhodobacteraceae sp. |  |  |  |
| 0.00 | **0.04** | Mesorhizobium |  |  |  |
| 0.03 | **0.04** | Schlegelella |  |  |  |
| 0.01 | **0.04** | Cupriavidus |  |  |  |
| 0.01 | **0.04** | Alcanivorax |  |  |  |
| 0.01 | **0.04** | Xenophilus |  |  |  |
| 0.00 | **0.04** | Pseudoxanthomonas |  |  |  |
| 0.00 | **0.04** | Cyanobacteria sp. |  |  |  |
| 0.00 | **0.04** | Rheinheimera |  |  |  |
| 0.00 | **0.04** | Dermacoccus |  |  |  |
| 0.01 | **0.03** | Achromobacter |  |  |  |
| 0.00 | **0.03** | Burkholderiales sp. |  |  |  |
| 0.01 | **0.03** | Rhodospirillaceae sp. |  |  |  |
| 0.01 | **0.03** | Enterobacteriaceae sp. |  |  |  |
| 0.00 | **0.03** | Mycobacterium |  |  |  |
| 0.00 | **0.03** | Epilithonimonas |  |  |  |
| 0.00 | **0.03** | Sandaracinobacter |  |  |  |
| 0.02 | **0.02** | Rhodocyclaceae sp. |  |  |  |
| 0.00 | **0.02** | Thiotrichaceae sp. |  |  |  |
| 0.00 | **0.02** | Escherichia-Shigella |  |  |  |
| 0.00 | **0.02** | Polynucleobacter |  |  |  |
| 0.00 | **0.02** | Sphingomonadaceae sp. |  |  |  |
| 0.00 | **0.02** | Terrimonas |  |  |  |
| 0.00 | **0.02** | Anaerolineaceae sp. |  |  |  |
| 0.01 | **0.02** | Undibacterium |  |  |  |
| 0.00 | **0.02** | Rhodococcus |  |  |  |

Relative abundance (%) that was significantly higher in the respective management group, is highlighted in bold. Significances were calculated on taxa occurring with 0.01% abundance within the whole dataset by applying non-parametric Kruskal-Wallis/FDR-P (alpha=0.05).
